# Measuring Navigational Health Literacy in Russia: Validation of the HLS_19_-NAV-RU

**DOI:** 10.3390/ijerph22020156

**Published:** 2025-01-24

**Authors:** Oxana Drapkina, Artemii Molosnov, Denis Tyufilin, Maria Lopatina, Viktor Medvedev, Valeriya Chigrina, Olga Kobyakova, Ivan Deev, Lennert Griese, Doris Schaeffer, Robert Griebler, Polina Tuillet, Anna Kontsevaya

**Affiliations:** 1 National Medical Research Center for Therapy and Preventive Medicine, 101000 Moscow, Russia; odrapkina@gnicpm.ru (O.D.); akontsevaya@gnicpm.ru (A.K.); 2 Russian Research Institute of Health, 127254 Moscow, Russia; molosnovam@mednet.ru (A.M.); tyufilinds@mednet.ru (D.T.); medvedevva@mednet.ru (V.M.); chigrinavp@mednet.ru (V.C.); kobyakovaos@mednet.ru (O.K.); 3 Department of Health and Life Sciences, Linnaeus University, 35252 Växjö, Sweden; 4 Ministry of Health of the Russian Federation, 127994 Moscow, Russia; deevian@minzdrav.gov.ru; 5 School of Public Health, Bielefeld University, 33615 Bielefeld, Germany; lennert.griese@uni-bielefeld.de (L.G.); doris.schaeffer@uni-bielefeld.de (D.S.); 6 Competence Centre for Health Promotion and Health System, Austrian National Public Health Institute, A-1010 Vienna, Austria; robert.griebler@goeg.at; 7 Institute of Leadership and Health Management, I.M. Sechenov First Moscow State Medical University, 109004 Moscow, Russia; tuillet_p_s@staff.sechenov.ru

**Keywords:** health literacy, navigational, questionnaire, validation, population, Russia

## Abstract

Structures and regulations of healthcare systems in many countries have become increasingly complex and difficult for patients and users to navigate. Thus, more than ever before, navigational health literacy (NAV-HL) is needed by patients. There are no data on NAV-HL in Russia due to the lack of suitable concepts and measuring tools. Therefore, the study aimed to validate the HLS_19_ navigational health literacy assessment tool (HLS_19_-NAV) for the Russian-speaking population. This study provides a comprehensive overview of the validation process, including a comprehensibility check and psychometric analysis. Overall, the results of the HLS_19_-NAV-RU validation demonstrate the partial validity of the NAV-HL tool in the Russian language. Nevertheless, the instrument can be recommended for further research and use in health literacy studies in Russia.

## 1. Introduction

In the last few decades, structures and regulations of healthcare systems in many countries, including the Russian Federation, have become increasingly complex and difficult for patients and users to navigate [1,2].

Navigating the healthcare system is an essential part of the process of obtaining timely and high-quality medical care and preventing various diseases. Moreover, as part of the transition from a paternalistic to a partnership model, patients are expected to be more actively involved in maintaining their health and to take more responsibility for their health and healthcare. This requires general health literacy (GEN-HL) and the ability to navigate the healthcare system, as well as relevant information. As a consequence, the navigational health literacy (NAV-HL) of patients is needed more than ever.

NAV-HL is defined as “people’s knowledge, motivation and skills to access, understand, appraise, and apply the information and communication in various forms necessary for navigating healthcare systems and services adequately to get the most suitable health care for oneself or related persons” [3].

The first population health literacy study in the Russian Federation was conducted within the Health Literacy Survey 2019–2021 (HLS_19_), a project of the WHO Action Network on Measuring Population and Organizational Health Literacy (M-POHL) conducted in 17 countries [4]. In the M-POHL HLS_19_ survey not only general HL was investigated [5], but also specific HLs, namely communicative HL with physicians, digital HL, vaccination HL, and navigational HL using newly developed and validated instruments [4,5,6].

In response to the lack of measurement tools and population-based data on NAV-HL, one of the aims of the M-POHL HLS_19_ project was to develop and introduce a concept-based instrument to measure NAV-HL using one and the same instrument in different countries for the first time [6]. The HLS_19_-NAV instrument was translated into seven languages: Czech, Dutch, French, German, Italian, Portuguese, and Slovenian [6,7]. The instrument for measuring NAV-HL was developed based on a scoping review of the literature, expert discussions, focus groups, and ongoing discussions in the HLS_19_ Consortium of M-POHL [3]. This resulted in a 12-item questionnaire measuring self-perceived ease or difficulty by a four-point rating scale (very easy, easy, difficult, and very difficult) in accessing, understanding, appraising, and applying navigation-related information at three different levels: system, organizational, and interactive levels [6].

Since Russia measured only GEN-HL within HLS_19_ [8] and did not include the NAV-HL package, there was no eligible tool for measuring NAV-HL in the Russian language. The aim of this study was to validate the HLS_19_-NAV questionnaire for the Russian-speaking population.

The study provides a comprehensive overview of the validation process, including the methodology used and the results obtained. It also discusses the various strategies applied to assess the credibility of the HLS_19_-NAV-RU questionnaire, including psychometric analysis and comprehensibility. The study also touches upon the problem of cultural adaptation of the newly developed tools, which is rather a relevant issue for non-English-speaking European countries [9] or non-European countries with different healthcare systems and historical backgrounds [10].

In addition, it provides challenges and limitations of validating the HLS_19_-NAV-RU tool and suggests recommendations for future research in this area.

## 2. Materials and Methods

The validation of the HLS_19_-NAV questionnaire [6] for the Russian population included the following steps:

### 2.1. Translation of the HLS_19_-NAV Questionnaire: Forward Translation from English into Russian and Backward Translation from Russian into English

#### 2.1.1. Forward Translation

The initial translation of the questionnaire from English to Russian was made by two independent translators to better reflect the nuances of the target language. In order to provide a translation that was closer to the original instrument, one of the translators was aware of the concept that the questionnaire intended to measure. The second translator was not aware of the underlying model and latent variable, which was to identify subtle differences with the original questionnaire. Discrepancies between these two translations were discussed and resolved with the help of an unbiased, bilingual translator who had not been involved in the previous translations.

#### 2.1.2. Backward Translation

To ensure the accuracy of the translation, the agreed forward translation was then independently translated from the target language back into the original language by two other independent translators, who were not aware of the intended concept of the questionnaire. The results showed minor variations in comparison with the original version only in terms of the use of synonyms.

### 2.2. Focus Group with the Target Population

Participants for both the focus group and the pre-test survey were recruited through a random selection process to minimize potential bias. The selection was conducted using a comprehensive list of insured individuals within the study’s target population. Randomization continued until the required sample size was achieved for each component of the study. This approach was designed to ensure that the sample was representative and to mitigate any systematic biases that might arise during the recruitment process.

The participants of the focus group (*n* = 10) were selected using a convenience sampling [11], which facilitated the participation of available respondents, who met the inclusion criteria: men and women, aged between 18 and 74, with no medical training [11].

Participants signed informed consent to personal data processing and answered the questions of the questionnaire, indicating whether the questions were clear or not. They were then asked to express their opinion on the structure, understandability, and content of the questionnaire.

### 2.3. Pre-Test Survey

The data for validating the questionnaire were collected via computer-assisted telephone interviews (CATI) in the Tula region of Russia, facilitated by the regional Ministry of Health. The assessment of construct (factorial) validity focused exclusively on the inter-item correlations of the questionnaire rather than the representativeness of the sample for the entire region. To achieve sufficient statistical power for the confirmatory factor analysis (CFA), a simple random sampling method was employed, ensuring efficient data collection. A total of 501 interviews were conducted, with 214 completed, yielding a response rate of 11.7%. Based on established guidelines [12], a sample size of 240 to 360 respondents is recommended to achieve at least 80% statistical power at a significance level of α = 0.05, depending on factor loadings and inter-variable correlations. Furthermore, Kline [13] suggests that latent variable models estimated using maximum likelihood require a sample size-to-parameter ratio (N:q) of 20:1 to ensure statistical precision and minimize technical problems in the analysis.

### 2.4. Statistical Analyses

A standard set of statistical methods was used to validate the HLS_19_-NAV-RU. Data were analyzed using the open-source statistical language R and the community-developed packages psych [14], EFA tools [15], and lavaan [16]. Non-normality issues inherently associated with the use of maximum likelihood estimations were controlled using bootstrapping library nFactors [17]. To clarify whether the data were suitable for factor analysis, the Kaiser–Meyer–Olkin (KMO) test, a measure of the proportion of variance between variables that could be common variance [18], and Bartlett’s test of sphericity, which compares the observed correlation of the matrix with the identity matrix, were performed [19].

The internal consistency of the scale was assessed by Cronbach’s alpha and Pearson item–total correlation. Cronbach’s alpha can be interpreted as the lower bound of the true internal consistency [20]. In the literature, a minimum value of alpha 0.7 is recommended [21]. The item–total correlation coefficients demonstrate the relationship between individual test item scores and the overall test score. In Likert-type scales, high positive correlations indicate strong internal consistency and effective measurement of the targeted concept, with coefficients above 0.20 considered acceptable [22]. The factor structure was evaluated in accordance with the original factor structure proposed by Griese et al. [3] with confirmatory factor analysis (CFA) without introducing modification indices or other methods of model refinement. The MLE extraction method was used.

The main purpose of conducting a CFA was to assess the adequacy of an assumed factor structure. Four fit indices were used to evaluate the quality of the obtained model:

- The Standardized Root Mean Square Residual (SRMR), which is the standardized difference between the observed and predicted covariance matrices in the model. It is calculated as the square root of the mean of the squared residuals, with values below 0.08 generally indicating an acceptable model fit [23];
- Comparative Fit Index (CFI), which evaluates the discrepancies between the hypothetical model and the actual data. Previously, the scientific community set the threshold for the CFI at ‘0.9 and above’, but Hu & Bentler [23] have shown that this threshold can be exceeded under certain conditions, including inadequate models, and therefore the value was raised to ‘0.95 and above’ [18]. A clear advantage of the CFI is its insensitivity to sample size, which makes it preferable to the chi-squared test;
- Tucker–Lewis Index (TLI), which estimates the difference between the chi-squared value in the hypothetical model and the null model, anticipating the possible biases of the Normed Fit Index. The threshold value was set at 0.90, as suggested by Byrne [24];
- Root Mean Square Error of Approximation (RMSEA), which is a standard in validation studies [24]. A threshold value is defined for both the RMSEA itself and its 90% confidence interval (CI) of no more than 0.08 at the upper limit.

Considering all these measures together allowed us to properly avoid both Type I and Type II errors.

## 3. Results

### 3.1. Focus Group

The results of the focus group showed that, in general, the questions were clear for the participants of the focus group, but some questions needed clarification, especially those related to health insurance, which is different in the Russian Federation compared to European countries. Also, the term ‘medical providers’ needed to be changed to match the characteristics of the Russian healthcare system, where they are referred to as ‘medical organizations’. All these inconsistencies were resolved, and the questionnaire was modified considering the respondents’ comments.

The main changes made in the questionnaire after putting together all the information collected via focus groups were as follows:

- Replacing the term “health insurance” with the term known to the wider Russian population “Polis OMS” (a card for compulsory medical insurance system serving as a proof of health insurance);
- Simplifying the term “healthcare institution” (provider) with “healthcare organization” due to the absence of legal private or any other forms of practice outside of healthcare organizations;
- Replacing the phrase “stand up for yourself” with “defend your right”, which sounds less confrontational and more suitable in the healthcare context.

### 3.2. Pre-Test Survey

#### 3.2.1. Descriptive Statistics and Data Adequacy

Based on our data, we calculated the descriptive statistics and the correlation matrix for the HLS_19_-NAV-RU items (Table 1). There are both high and low correlations in the matrix, indicating that the factor structure of the HLS_19_-NAV-RU may be more complex than a single-factor solution. However, considering the number of low inter-item correlations, we needed to determine whether our data were ‘suited’ for further analysis.

The overall KMO was 0.85, indicating a good fit of the data for factor analysis. Bartlett’s sphericity test [25] was significant at the *p* < 0.001 level (*χ*^2^ (66) = 1471.41), which assures us that the data are suitable for factor analysis.

#### 3.2.2. Internal Consistency

Before conducting factor analysis, we checked the internal consistency of the HLS_19_-NAV-RU using Cronbach’s alpha and item–total correlations (Table 2). Cronbach’s alpha for the total scale was 0.85, and it showed little sensitivity to excluding any of the items from the scale (α from 0.83 to 0.85). Thus, according to common views on alpha thresholds, the scale had a more than acceptable level of internal consistency [25]. However, we should note the low item–total correlation of q7 (find information on the quality of a particular health service) and the fact that internal consistency does not decrease with the removal of this item.

#### 3.2.3. Confirmatory Factor Analyses

A single-factor solution was tested, which showed poor metrics (RMSEA = 0.090, Comparative Fit Index (CFI) = 0.867, Tucker–Lewis Index (TLI) = 0.837), only the Standardized Root Mean Square Residual (SRMR) equals 0.063 (assuming SRMR (SRMSR) ≤ 0.08 was adequate to the model).

**Figure 1:**
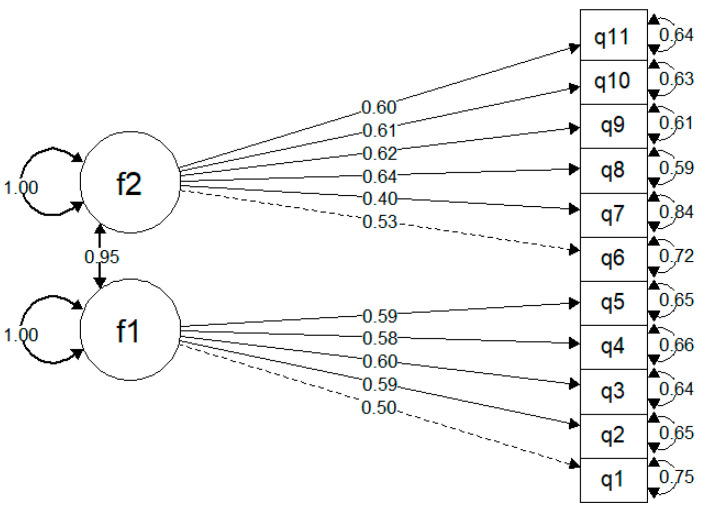
Confirmatory factor analysis (2-factor model). The circles represent latent factors, while the rectangles correspond to observed variables. The lines connecting the latent factors to the observed variables are standardized factor loadings. Dot lines indicate fixed parameters used for scaling by fixing factor loadings. The arrow between the two latent factors represents the covariance of the latent constructs. Arrows from the latent factors to themselves denote the latent factor variances, and arrows from the observed variables to themselves represent the variances of the observed variables.

Testing a two-factor model taken from Griese et al. [3], we obtained SRMR = 0.059, and RMSEA = 0.080, indicating a better fit (Figure 1). However, the CFI and TLI equal 0.895 and 0.866, respectively, remaining below the accepted thresholds of 0.9 or 0.95 (Table 3).

The considerations for testing the factor structure are based on Griese et al. (2020; 2022). The authors showed that when fitting a single-factor CFA, the HLS_19_-NAV data obtained acceptable goodness-of-fit indices across HLS_19_ countries. However, as the HLS_19_-NAV is based on a framework with theoretically derived levels (system, organization, and interactive level), a multidimensional solution was also proposed, which was confirmed by a two-factor CFA model (items HLS_19_-NAV1-5, items HLS_19_-NAV6-11), showing improved fit values in comparison to the single-factor model (Griese et al., 2022).

In regard to the HLS_19_ results, a single-factor solution was tested for the Russian data, which showed poor metrics (RMSEA = 0.090, Comparative Fit Index (CFI) = 0.867, Tucker–Lewis Index (TLI) = 0.837). Only the Standardized Root Mean Square Residual (SRMR) equals 0.063 (assuming SRMR (SRMSR) ≤ 0.08 was adequate to the model).

Testing the two-factor model taken from Griese et al. [3], we obtained SRMR = 0.059, and RMSEA = 0.080, indicating a better fit. However, the CFI and TLI equal 0.895 and 0.866, respectively, remaining below the accepted thresholds of 0.9 or 0.95 (Table 3).

**Figure 2:**
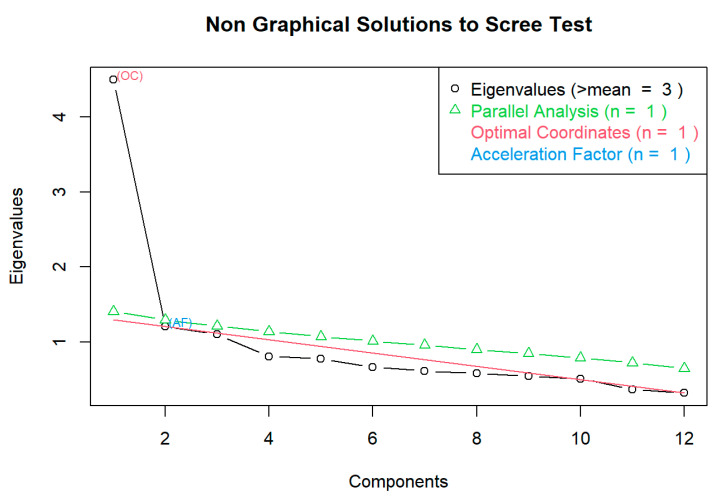
Scree plot. The circles represent the eigenvalues calculated from the data. The green triangles indicate the results of parallel analysis, which compare the observed eigenvalues with those generated from random data. The red line labeled “OC” marks the optimal coordinates, which corresponds to an extrapolation of the preceding eigenvalue by a regression line between the eigenvalue coordinates and the last eigenvalue coordinates [17]. The blue dot labeled “AF” represents the acceleration factor. These methods identify the number of components to retain in a factor analysis. The analysis was conducted using Spearman correlations, with 1000 random data replications for parallel analysis at the 95th percentile quantile.

Therefore, the results of the HLS_19_-NAV-RU validation demonstrate good internal consistency and partial validity of the tool in the Russian language. Additionally, a scree plot suggests the two factors, as the eigenvalue stays relatively the same only after the 2nd factor (Figure 2) [26].

## 4. Discussion

This study is the first one in the Russian Federation that is concerned with NAV-HL. For this purpose, the HLS_19_-NAV questionnaire was first used for the Russian population.

The results of the validation study of the HLS_19_-NAV-RU partially support the construct validity of the tool, showing a partially acceptable fit to the model tested by Griese et al. [6]. The factor structure was evaluated in accordance with the original factor structure proposed by Griese et al. [3] with confirmatory factor analysis (CFA). A two-factor model showed a better (but still not satisfactory) fit compared to the one-factor model. This may indicate either slight difficulties in the cultural adaptation of the HLS_19_-NAV-RU or mild differences between the Russian Federation and the eight European countries studied in Griese et al. [6] in terms of how information is provided in different healthcare systems. Nevertheless, judging from the final factor model of NAV-HL, the item’s functions are assigned in a similar way compared with Griese et al. [3].

Out of 12 items of the questionnaire, the only deviation from the proposed 2-factor model was found for item q7 (*to find information on the quality of a particular health service*), which did not load sufficiently on the corresponding system-level factor (estimate 0.4 compared to 0.5–0.6 for other items). This item also showed a poor fit for the one-factor model. Indeed, in the Russian context, information about the quality of healthcare and, especially, particular services is not available to recipients of care. The usual ways people find this information on the quality of health services is a search for other patients’ reviews that might be rather subjective and not always constructive, as well as personal conversations, and unofficial social media coverage. At the same time, due to the lack of official criteria and standards for healthcare services quality (both state and private), many patients rely on their own lay criteria for evaluation of the quality of healthcare services [27,28,29].

Taken together, respondents probably did not consider the option of finding information on the quality of health service as it is not officially and publicly available information.

In addition, we did not observe any response dependency between items, as Griese et al. [6] found for some countries with questions q7 (find information on the quality of a particular health service) and q8 (judge if a particular health service will meet your expectations and wishes on healthcare), indicating that items have something in common and intend to measure related aspects that are important for choosing a particular health service. No response dependency in our survey suggests that due to cultural specifics patients in Russia might have their own perceptions and expectations about the outcome of interaction with a healthcare system, which might not be associated with the availability of information provided and criteria of the quality of particular health services.

The study of NAV-HL in eight European countries revealed that HL-NAV is determined by sociodemographic and socioeconomic factors, as was shown for general HL [6]. NAV-HL was also linked to general health status in most countries, indicating that the measure is able to provide some kind of predictive value [6].

Also, the relationship between NAV-HL and GEN-HL was studied, and concluded that HLS_19_-NAV added new aspects in managing health information for the specific context of navigating different healthcare systems [6].

Unfortunately, due to the fact that GEN-HL and NAV-HL were studied in Russia separately, it would be reasonable to study NAV-HL together with GEN-HL in order to identify associations and influencing factors on both measures.

Based on the obtained results, it could be suggested for further research, due to cultural specifics of navigating the healthcare information through the interaction in Russia, to include more items and have a deeper study of NAV-HL at the interactive level, which was also updated in the follow-up study HLS_24_, to improve the content validity of the instrument. Until now, the interactive level is represented by only one item (stand up for yourself if your healthcare does not meet your needs), though it is more likely that interacting with health services and professionals is important for navigating the healthcare system.

### Limitations of the Study and Suggestions for Future Research

One limitation of the study is that it was conducted only in one region of the Russian Federation and that the sampling was not representative of the chosen region, which may limit the generalizability of the results [30]. The low response rate (11.7% for complete data points) may result in non-response bias since respondents may be inherently different from non-respondents. There were also more incomplete interviews than complete ones, resulting in the loss of potentially important data, which could also bias the results, especially if the reasons for noncompletion are correlated with study variables. In addition, we observe the rare use of extreme response categories, which could be due to both the method of data collection and the proposed response options. Moreover, the tool validation process is only concerned with the correlations between the items of the questionnaire and not with the survey results as such. Also, it is important to point out that the study was not aimed at making the generalization of the results to the Russian Federation as a whole; it would be the purpose of next studies. Moreover, in terms of NAV-HL, it seems reasonable to have results for each separate region, taking into consideration the context of every region in particular.

Another limitation is that the respondents’ answers may be based not only on the respondents’ direct experiences, but also on their general perceptions. This is a known problem with self-reporting, but when compared to objective measures, it provides an opportunity to better understand HL barriers from a user’s perspective.

## 5. Conclusions

Navigating the healthcare system is essential for obtaining timely and high-quality medical care and preventing diseases. Moreover, as part of the transition from a paternalistic to a partnership, patient-centered model, patients are expected to be more actively involved in maintaining their health and to take more responsibility for their health and healthcare. Therefore, measuring NAV-HL allows for identifying barriers to patients’ navigation of the healthcare system and finding the solutions to overcome those barriers.

The translation, adaptation, and validation of the HLS_19_-NAV-RU is an important step towards measuring NAV-HL, in general, the adult population in Russia.

The results of the HLS_19_-NAV-RU validation demonstrate good internal consistency and only a partial validity of the tool in the Russian language presumably due to cultural specifics on managing health-related information.

The study demonstrated that would be preferable to study NAV-HL together with GEN-HL and more in-depth at the interactive level due to ways of accessing healthcare information and cultural aspects in its operation.

Further comprehensive research is needed to continue refining and improving the tool, including interactive skills important for navigating the healthcare system. In conclusion, the instrument can be recommended for further research in a representative sample for each region of the RF and use in health literacy studies in Russia.

### Instrument Use

The instrument belongs to the HLS_19_ Consortium. The use of the instrument needs a contractual agreement between a non-profit applicant and the HLS_19_ Consortium. More information on the use of the instrument can be found here: https://m-pohl.net/HLS19Instruments URL (accessed on 4 December 2024).

## Figures and Tables

**Table 1:** HLS_19_-NAV-RU item correlations mean and standard deviation.

	q1	q2	q3	q4	q5	q6	q7	q8	q9	q10	q11	q12
q1	–											
q2	0.280	–										
q3	0.320	0.590	–									
q4	0.370	0.220	0.310	–								
q5	0.230	0.300	0.280	0.360	–							
q6	0.260	0.220	0.220	0.350	0.270	–						
q7	0.140	0.170	0.200	0.190	0.350	0.080	–					
q8	0.350	0.340	0.290	0.340	0.350	0.270	0.300	–				
q9	0.270	0.360	0.220	0.260	0.350	0.280	0.290	0.430	–			
q10	0.350	0.320	0.380	0.380	0.270	0.430	0.110	0.340	0.300	–		
q11	0.420	0.230	0.250	0.380	0.320	0.360	0.190	0.360	0.380	0.400	–	
q12	0.400	0.250	0.250	0.410	0.390	0.330	0.150	0.430	0.320	0.440	0.630	–
Mean	2.56	2.74	2.71	2.29	2.69	2.35	2.94	2.66	2.81	2.19	2.40	2.16
SD	0.60	0.54	0.54	0.63	0.56	0.65	0.50	0.55	0.51	0.66	0.63	0.72

q1: understand information on how the healthcare system works, e.g., which type of health services are available; q2: judge which type of health service you need in case of a health problem; q3: judge to what extent your health insurance covers a particular health service, e.g., are there any co-payments; q4: understand information on ongoing healthcare reforms that might affect your healthcare; q5: find out about your rights as a patient or user of the healthcare system; q6: decide for a particular health service, e.g., choose from different hospitals; q7: find information on the quality of a particular health service; q8: judge if a particular health service will meet your expectations and wishes on healthcare; q9: understand how to get an appointment with a particular health service; q10: find out about support options that may help you to orientate yourself in the healthcare system; q11: locate the right contact person for your concern within a healthcare institution, e.g., in a hospital; q12: stand up for yourself if your healthcare does not meet your needs.

**Table 2:** Item total correlation and Cronbach’s alpha for the scale.

Item	Pearson Item–Total Correlation	Alpha Without Item
q1	0.47	0.84
q2	0.50	0.84
q3	0.51	0.84
q4	0.54	0.84
q5	0.55	0.84
q6	0.48	0.84
q7	0.34	0.85
q8	0.58	0.84
q9	0.55	0.84
q10	0.56	0.84
q11	0.59	0.83
q12	0.60	0.83

**Table 3:** CFA: Model comparison based on goodness-of-fit indices.

Model	SRMR	RMSEA	RMSEA Upper 90% CI	RMSEA Lower 90% CI	CFI	TLI
1—factor	0.063	0.090	0.107	0.073	0.867	0.837
2—factor	0.059	0.080	0.101	0.061	0.895	0.866

## Data Availability

The original contributions presented in this study are included in the article. Further inquiries can be directed to the corresponding author(s).
